# Metagenome-assembled genomes of *Macrocystis*-associated bacteria

**DOI:** 10.1128/mra.00715-24

**Published:** 2024-10-22

**Authors:** Emily G. Aguirre, Julia A. Schwartzman

**Affiliations:** 1 Department of Biological Sciences, University of Southern California, Los Angeles, California, USA; 2 AltaSeads Conservancy, San Pedro, California, USA; DOE Joint Genome Institute, Berkeley, California, USA

**Keywords:** kelp, host–microbe interaction, metagenome-asssembled genome, *Macrocystis*, marine microbiology, microbiome

## Abstract

Microbes are important for the development of kelp, but little is understood about the functions encoded by these microbes. Here, we assembled 1,794 metagenome-associated genomes (MAGs) from bacteria that colonize gametophytes of the giant kelp *Macrocystis pyrifera*. MAGs were grouped into 149 dereplicated strains. Functional annotation is also presented.

## ANNOUNCEMENT

Microbes affect the recruitment and growth of the giant kelp, *Macrocystis pyrifera* (1–3), an ecologically and economically relevant brown macroalgae (4, 5). However, the microbial ecology of *M. pyrifera* gametophytes (early-life stage) remains understudied (1, 2). To better understand the metabolic potential of taxa frequently associated with gametophytes, we constructed metagenome-assembled genomes (MAGs) from publicly available short-read sequencing of *M. pyrifera* gametophytes.

Short-read sequencing of 178 individual *M. pyrifera* gametophytes were retrieved from National Center for Biotechnology Information Sequence Read Archive (NCBI SRA; PRJNA1050779). As described elsewhere (2), reads came from total DNA extracted from individual 3-month-old gametophytes generated from mature blades originating from four sites along the Southern California coast. DNA was extracted from gametophyte biomass using the NucleoSpin 96 Plant Kit (Macherey-Nagel), and samples were sequenced on an Illumina S4 NovaSeq platform (2 × 150 bp reads).

Reads were trimmed using FastP v0.23.4 (6), which removed low-quality sequences and adapters. Reads mapping to PhiX, *M. pyrifera*, and human contaminant DNA were removed using bbduk.sh in BBmap v38.16 (7), with custom masks for human (8) and *M. pyrifera*, using the reference genome (9), accession PRJNA926673. The remaining reads (average per gametophyte 1.8 * 10^9^, minimum = 6.9 * 10^7^ maximum = 3.4 * 10^9^) were error-corrected using BayesHammer, part of SPADES v3.15.5. *De novo* assemblies were generated for each gametophyte sample using MEGAHIT v1.2.9 (10), resulting in contigs that were binned using MaxBin2 v2.2.7 (11) and CONCOCT v1.0.0 (12). Non-redundant bins (MAGs) were generated using DASTool v1.1.6 (13). MAG quality was then assessed with CheckM2 v1.0.1 (14). Additional genome curation was performed using NCBI’s Foreign Contamination Screen (15).

To identify assemblies across samples that shared >95% genome-wide average nucleotide identity (ANI), MAGs estimated as >90% complete and <10% contaminated were filtered. Taxonomy for the whole data set was assigned using classify_wf in GTDB-Tk v2.3.2 (16) . Resulting alignments were used as input for FastTree v2.1.11 to produce a maximum likelihood phylogeny and visualize taxonomic diversity. MAGs were dereplicated using dRep v3.4.4 (17), and this set was annotated by predicting protein-coding genes using the -p meta flag on Prodigal v2.6.3 (18), and assigning functions using the METABOLIC-G.pl function on METABOLIC v4.0 (19).

**FIG 1:**
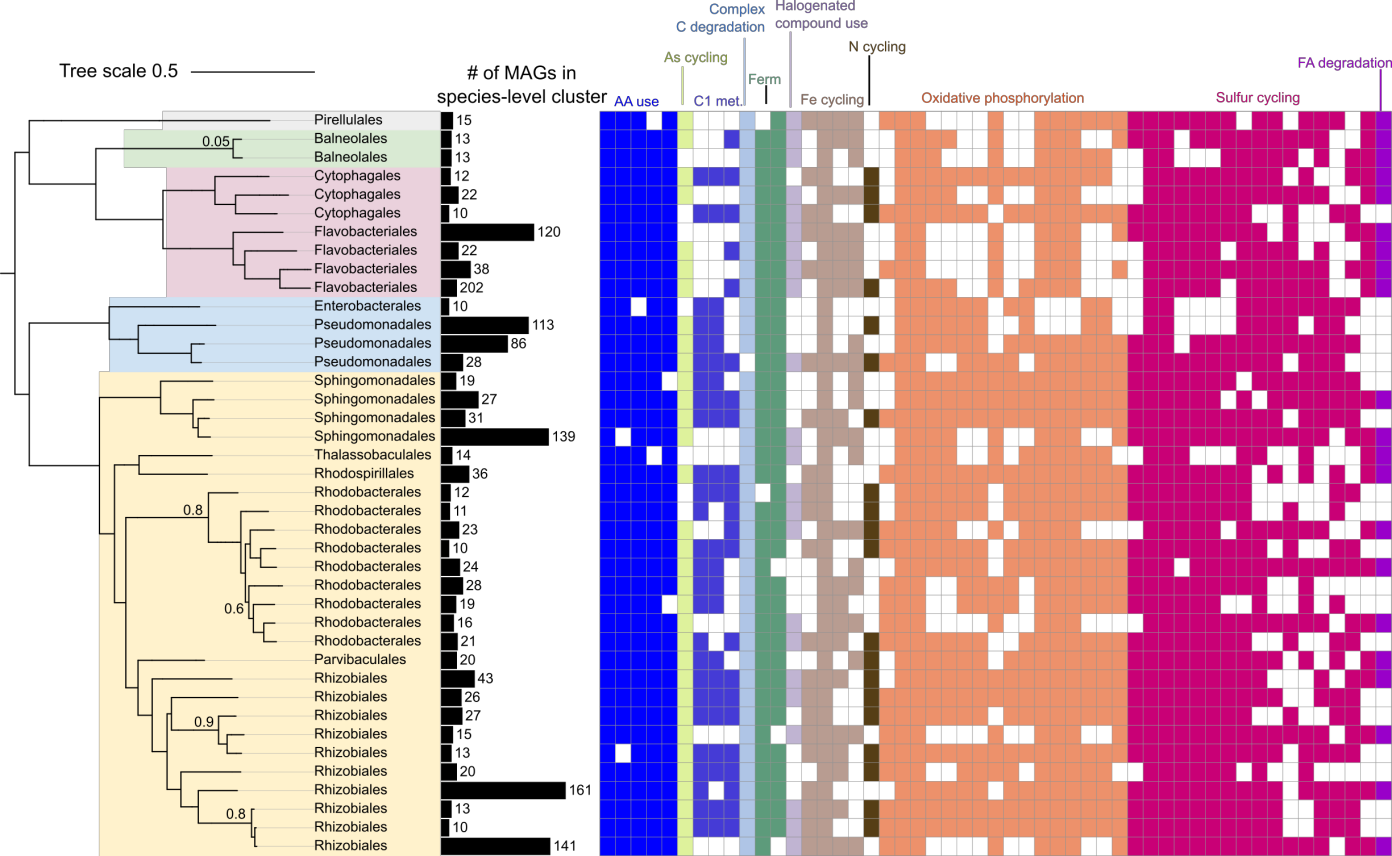
Phylogeny of MAGs assembled at least 10 independent times from gametophyte metagenomes. Approximately maximum-likelihood phylogeny with bootstrap (*n* = 100), built using concatenated marker proteins from the GTDB-Tk “Classify” function output and generated with FastTree v2.1.11. Labels indicate the predicted taxonomic order of each MAG, and colored ranges indicate the predicted taxonomic class. The tree was pruned and visualized using iTOL (20). Bar graph indicates the number of assemblies of a MAG sharing >95% average nucleotide identity from independent gametophyte metagenomes. Heatmap indicates pathways fully or partially present in each MAG, as predicted by METABOLIC (18).

From the 178 gametophyte samples, we assembled 1,794 MAGs (Table 1). The dereplication of this set to 149 MAGs indicates that a large set of MAGs assembled from different gametophytes shared >95% ANI. Taxonomic diversity in MAGs spanned 5 phyla and 19 orders. We quantified the number of independent assemblies represented by each dereplicated MAG. A quarter of the MAGs were assembled 10 or more times (Fig. 1). METABOLIC predictions of encoded gene function for this set of 40 MAGs revealed that metabolic pathways, including sulfur oxidation, respiration of acetate, chitin decomposition, catabolism of amino acids, and arsenate reduction, are widely encoded in the genomes of bacteria that commonly colonize gametophytes (Fig. 1).

**TABLE 1:** Summary of MAGs assembled in this study^*a*^

Taxonomic classification	#^*b*^	% Gametophytes^*c*^	Average % GC^*d*^	Average genome size (Mb)^*e*^
*Algibacter* sp.	6	3.4	32	4.67
*Algibacter* sp*.*	1	0.6	33	3.88
*Algoriphagus* sp*.*	9	5.1	42	4.45
*Aliiglaciecola* sp*.*	1	0.6	41	4.92
*Aliishimia* sp*.*	1	0.6	54	5.89
*Alloalcanivorax* sp*.*	1	0.6	58	3.72
*Alloalcanivorax venustensis*	8	4.5	65	3.44
*Alphaproteobacteria* bacterium	36	20.2	63	3.93
*Alphaproteobacteria* bacterium	2	1.1	63	3.71
*Alteripontixanthobacter* sp*.*	3	1.7	62	2.82
*Anderseniella* sp*.*	43	24.2	56	4.76
*Ascidiaceihabitans* sp*.*	19	10.7	55	4.21
*Aurantibacter* sp*.*	1	0.6	34	4.51
*Aurantimonas coralicida*	2	1.1	67	3.98
*Balneola* sp*.*	14	7.9	37	4.07
*Balneola* sp*.*	13	7.3	38	3.53
*Balneola* sp*.*	1	0.6	37	3.09
*Bauldia litoralis*	6	3.4	66	5.04
*Cellulophaga* sp*.*	1	0.6	32	3.77
*Cobetia amphilecti*	1	0.6	62	4.24
*Crocinitomicaceae* bacterium	7	3.9	36	4.08
*Cyclobacteriaceae* bacterium	10	5.6	43	5.23
*Cyclobacteriaceae* bacterium	6	3.4	43	5.21
*Cyclobacteriaceae* bacterium	1	0.6	42	4.92
*Cyclobacteriaceae* bacterium	1	0.6	41	4.87
*Dokdonia* sp*.*	3	1.7	34	5.31
*Ekhidna* sp*.*	36	20.2	38	4.94
*Ekhidna* sp*.*	22	12.4	41	4.69
*Ekhidna* sp*.*	2	1.1	40	4.33
*Ekhidna* sp*.*	1	0.6	39	4.28
*Erythrobacter* sp*.*	6	3.4	54	2.86
*Erythrobacter* sp*.*	5	2.8	56	3.13
*Erythrobacter* sp*.*	4	2.2	55	3.30
*Erythrobacter* sp*.*	3	1.7	51	3.16
*Erythrobacter* sp*.*	2	1.1	56	2.73
*Erythrobacter* sp*.*	1	0.6	56	3.19
*Erythrobacter* sp*.*	1	0.6	56	3.28
*Eudoraea* sp*.*	3	1.7	37	3.92
*Flavobacteriaceae* bacterium	20	11.2	39	4.20
*Fuerstiella* sp*.*	1	0.6	51	7.44
*Fulvivirga* sp*.*	1	0.6	39	4.27
*Gillisia* sp*.*	1	0.6	40	3.57
*Gilvibacter* sp*.*	22	12.4	40	3.27
*Halieaceae* bacterium	2	1.1	56	3.93
*Halieaceae* bacterium	2	1.1	58	4.24
*Halioglobus* sp*.*	3	1.7	53	3.93
*Halioglobus* sp*.*	2	1.1	48	4.24
*Halioglobus* sp*.*	1	0.6	53	3.73
*Halioglobus* sp*.*	1	0.6	54	4.64
*Haloferula* sp*.*	2	1.1	58	4.83
*Hellea* sp*.*	1	0.6	49	4.04
*Henriciella* sp*.*	2	1.1	55	3.64
*Hoeflea* sp*.*	1	0.6	60	4.91
*Hyphomicrobiales* bacterium	26	14.6	50	4.42
*Hyphomicrobiales* bacterium	7	3.9	47	2.51
*Hyphomicrobiales* bacterium	3	1.7	48	3.30
*Hyphomicrobiales* bacterium	1	0.6	42	3.48
*Hyphomicrobiales* bacterium	1	0.6	48	3.33
*Hyphomonadaceae* bacterium	1	0.6	55	4.02
*Hyphomonas* sp*.*	8	4.5	60	4.05
*Ilumatobacter* sp*.*	6	3.4	68	4.68
*Kangiellaceae* bacterium	9	5.1	41	4.91
*Lentilitoribacter* sp*.*	140	78.7	44	3.73
*Lentilitoribacter* sp*.*	13	7.3	45	3.81
*Lentilitoribacter* sp*.*	10	5.6	44	3.96
*Litoreibacter* sp*.*	3	1.7	56	3.49
*Litoreibacter* sp*.*	1	0.6	52	5.30
*Litorimonas* sp*.*	3	1.7	50	3.26
*Litorimonas* sp*.*	1	0.6	46	2.72
*Luteolibacter* sp*.*	8	4.5	58	4.00
*Maribacter dokdonensis*	38	21.3	36	4.29
*Maribacter litoralis*	1	0.6	36	4.46
*Maribacter* sp*.*	1	0.6	35	4.22
*Maribacter* sp*.*	1	0.6	35	4.03
*Maribacter* sp*.*	1	0.6	39	4.58
*Maricaulaceae* bacterium	1	0.6	47	2.71
*Maricaulis* sp*.*	2	1.1	65	3.57
*Marinobacter alexandrii*	29	16.3	57	4.20
*Marinobacter* sp*.*	86	48.3	57	4.48
*Marinobacter* sp*.*	4	2.2	55	3.48
*Marinobacter* sp*.*	4	2.2	57	4.84
*Marinomonas* sp*.*	114	64.0	56	2.92
*Marinomonas* sp*.*	114	64.0	56	2.92
*Marinomonas* sp*.*	2	1.1	42	3.35
*Marinomonas* sp*.*	1	0.6	42	3.55
*Nisaea* sp*.*	14	7.9	59	5.15
*Nitratireductor* sp*.*	161	90.4	65	4.61
*Nonlabens* sp*.*	4	2.2	35	3.03
*Nonlabens ulvanivorans*	122	68.5	35	3.17
*Paracoccaceae* bacterium	28	15.7	53	4.56
*Paracoccaceae* bacterium	12	6.7	55	4.15
*Paracoccaceae* bacterium	11	6.2	54	5.13
*Paracoccaceae* bacterium	3	1.7	53	4.93
*Paracoccaceae* bacterium	3	1.7	53	4.91
*Paraglaciecola* sp*.*	10	5.6	40	4.43
*Paraglaciecola* sp*.*	2	1.1	39	4.13
*Paraglaciecola* sp*.*	1	0.6	41	5.67
*Parasphingorhabdus* sp*.*	140	78.7	53	3.48
*Parasphingorhabdus* sp*.*	48	27.0	54	3.34
*Parasphingorhabdus* sp*.*	31	17.4	53	3.58
*Parasphingorhabdus* sp*.*	7	3.9	50	3.90
*Parasphingorhabdus* sp*.*	3	1.7	51	3.44
*Parasphingorhabdus* sp*.*	1	0.6	54	2.92
*Parvibaculum* sp*.*	21	11.8	60	3.81
*Parvibaculum* sp*.*	6	3.4	64	3.73
*Planktotalea* sp*.*	1	0.6	54	4.11
*Polaribacter* sp*.*	3	1.7	29	3.29
*Porticoccus* sp*.*	1	0.6	53	2.76
*Pseudophaeobacter* sp*.*	4	2.2	59	4.52
*Pseudophaeobacter* sp*.*	1	0.6	57	4.99
*Pseudophaeobacter* sp*.*	1	0.6	58	4.39
*Pseudophaeobacter* sp*.*	1	0.6	59	4.17
*Pseudoruegeria* sp*.*	4	2.2	52	4.85
*Pyruvatibacter* sp*.*	2	1.1	58	3.56
*Qipengyuania citrea*	19	10.7	64	3.09
*Reichenbachiella* sp*.*	35	19.7	48	5.41
*Reichenbachiella* sp*.*	2	1.1	39	4.68
*Reichenbachiella* sp*.*	2	1.1	40	4.92
*Reichenbachiella* sp*.*	1	0.6	41	4.86
*Rhizobiaceae* bacterium	20	11.2	55	4.85
*Rhizobiaceae* bacterium	4	2.2	54	5.02
*Rhodopirellula bahusiensis*	15	8.4	56	7.63
*Rhodothermales* bacterium	1	0.6	69	4.60
*Roseibium album*	1	0.6	56	6.93
*Roseibium polysiphoniae*	1	0.6	58	5.48
*Roseibium* sp*.*	27	15.2	57	4.77
*Roseibium* sp*.*	15	8.4	62	5.71
*Roseibium* sp*.*	14	7.9	52	5.02
*Roseibium* sp*.*	2	1.1	53	5.03
*Roseobacter* sp*.*	2	1.1	51	3.77
*Saccharospirillum* sp*.*	5	2.8	56	4.66
*Sedimentitalea* sp*.*	4	2.2	58	5.85
*Shimia* sp*.*	1	0.6	56	4.73
*Shimia thalassica*	4	2.2	56	4.66
*Sneathiella* sp*.*	7	3.9	50	4.60
*Stappiaceae* bacterium	2	1.1	53	6.29
*Sulfitobacter dubius*	1	0.6	60	4.01
*Sulfitobacter pontiacus*	24	13.5	59	3.77
*Sulfitobacter* sp.	10	5.6	61	3.97
*Sulfitobacter* sp*.*	5	2.8	57	3.59
*Sulfitobacter* sp*.*	2	1.1	56	3.90
*Sulfitobacter* sp*.*	1	0.6	53	6.40
*Tateyamaria* sp*.*	21	11.8	56	3.97
*Tateyamaria* sp*.*	16	9.0	57	4.63
*Tateyamaria* sp*.*	3	1.7	57	4.43
*Vibrio splendidus*	1	0.6	44	5.56
*Winogradskyella arenosi*	3	1.7	44	3.59
*Yoonia* sp*.*	6	3.4	55	3.75

^*a*^ Full metadata available: Schwartzman, J. and Aguirre, E. (2024). Metadata for bacterial MAGs from giant kelp gametophytes [Data set]. Zenodo. https://doi.org/10.5281/zenodo.13851776.

^*b*^ Number of MAGs in cluster (assemblies sharing 95% or greater average nucleotide identity).

^*c*^ Percentage of gametophytes from which the MAG species-level cluster was assembled.

^*d*^ Average percent GC content for assemblies in this MAG species-level cluster.

^*e*^ Average genome size in megabases for assemblies in this MAG species-level cluster.

## Data Availability

MAG sequences and short-read sequencing data are available under the NCBI project accession PRJNA1050779. Scripts used to generate the MAGs, the functional analysis output, and detailed information about each MAG and its associated gametophyte are available at GitHub.
